# Microscopy of Minute Organisms

**Published:** 1872-08

**Authors:** S. P. Cutler


### SELECTED ARTICLES.

### ARTICLE III.

### *Microscopy of Minute Organisms.*

BY S. P. CUTLER, M.D., D.D.S.

Read before the New Orleans Academy of Science.

(CONCLUDED.)

Twenty-three specimens of mercurial preparations, mostly insoluble, all full of them; the cyanide, the most soluble, contained the fewest.

A specimen of bone from a skeleton of a poor girl that died in the Charity Hospital, of hereditary syphilis, was full of them.

Specimens of blood and black vomit from a patient that died at the hospital, contained them.

Several specimens of dead bone from fractured pieces, and pus running from the same, contained them; few in the pus, but myriads in the bone.

Fresh curd from cow's milk was intensely colonized with them, though no *oidium-lactis*—but large globules of various sizes were present.

A solution of tainted glue was full of them, only their actions were not so great, owing, no doubt, to the effect of gluten mechanically acting on their motions; besides these three, a number of *rotifera vulgaris*, apparently feeding on the others—*leptothrix* and *oidium albicans* were also seen.

Boiled starch, after standing four or five days, does not present them distinctly—only a nebulous cloud and starch cells, round, and varying in size.

After standing four weeks, until it becomes mouldy on the surface, it gave these forms in great abundance, besides the forms peculiar to such moulds.

Different specimens of beans, green corn in cans, oysters —all in abundance.

Sour clabber, a week old, full; also some *oidium lactis* and starch cells, from 1-8,000th to 1-2,000th of an inch in diameter, and some *oidum tomla—homicium*, or necklace pencilium beads, in fingered branches.

Condensed milk, bologna sausage, liver—full.

I put some of the milk on a piece of tin, and held it over the lamp until the milk was all charred to a coal by heating red hot; this burnt matter was completely composed of them, and perfectly vigorous and active.

Various kinds of pickles in the strongest vinegar, mustard seed, turnips—all full.

Vaccine matter, spinal marrow of chicken, specimens of toilet and shaving soap, cockroaches, mosquitoes, flies, bone cartilage, rotten flooring plank and other rotten wood, oxide of zinc, extract of (so called,) coffee, pieces of fresh meat kept five days in chloroform vapor, and dried, all contained them in various quantities. The predominant forms seen in all the above specimens were chiefly the small minute globular (or nearly so) forms.

I will make a few quotations from Dr. Theodore C. Hillgard's valuable pamphlet on *Infusorial Circuit of Generation*, and represented in the plate of illustrations. Dr. H. says; "The center 8; the vibrion truly fermentic element, awaiting the lactic, alcoholic, and vadaverous fermentations, and afterwards generating the yeast cells, from which these elements are again disgorged when opportunity is given." Old, crusty, and macerated fragments, re-dissolving into the naked vibratile molecules.

"I examined a great number of specimens of pollen from different flowers, and all contain them in great abundance. They may sometimes be seen coming out of the pollen heads, from a minute opening, like human beings from a church on Sundays; this is when they are ripe. It is better to mash them on the glass for examination. These pollen, or seed vessels, are carried on the feet of insects from one plant to another, and in this way many flowers are fructified that otherwise would not be; the wind, also, blows them about; and who shall say that these little organisms have no agency in the process of fecundation in plants as in animals. I am quite convinced that all kinds of pollen contain them, also all kinds of vegetable growth, varying only in quantities, the starch containing less than any other organic vegetable compounds of nutritive plants. I am also convinced that all animal substances contain them in abundance, varying in different animals, or fermentic vibrions. The latter, by lengthwise inosculation, are seen to connect singly, or by pairs, string into the delicate naked vibrionic single file, threads or .leptothrix, forming short weltering curves (churnus,) and long rounding archimedian spirals, with spinning butt ends."

I had but few of the latter in specimens from the mouth and specimens of spoiled glue.

Further on Dr. H. says: "Figure 11 represents the yeast cells elongated into micelium, which segments into cylinder fragments, *oidium-lactis*, which at the same time rearing its penciled floritions and mucurine seed vessels on one identical fiber with *oidium.* Some submerged pencils, and also some turgid segments, pseudo *zooglina*, are seen emitting coted bacteria, which reproduce the finest *leptomitus* fiber. The latter as *leptomitus bacterius*, again digests into globular individual molicular germs—1000 to 2000 lines diameter."

The writer may be right in these statements, but the last I have not been able to see, though I have not studied the process sufficiently to pass a decided opinion in the negative.

My chief object in prosecuting these researehes is to determine the habitudes and habitants of these, the almost omnipresent organisms, the smallest units of ontology, and to endeavor to show their probable agency in physiological and pathological conditions, their connection with contagion and death, their cause, and consequence of all fermentative action, though they never themselves become elevated with anything higher, as the *homicium*, detached joints, or in the form of necklace yeast, or the round globules, or *cryptococus*, of the authors.

The author referred to further says: "The lactic fermentation of sweet milk into clabber, as well as corn-meal mashes at a low temperature, is often completely enacted without any other form of yeast fungus but its natural vibrias and vibrioni *churnis*, the incessant motion of the latter, which, under a few hundred magnifying powers are barely discernable to a practical eye."

Here the author recognizes the same organisms I have been describing, only he confines them to sweet milk. I find them everywhere there is or has been life or organic matter, and everywhere else.

Dr. H. says further, on page 272:

"Simple naked fermentic sarcode vibrios, of a globular shape, 1-6,000 line or less in diameter, with a viratory organ not distinctly discernable, but causing a salutatory motion or jerks, often ten fold their diameter. Frequent experiments have satisfied me that without the presence of any other (cell or sarcode) development, they are by themselves sufficient to enact the entire alcoholic, lactic and putrid (cadaverous,) fermentation, or decomposition, without aid from other organs."

These salutary motions, above mentioned, are truthfully rendered where mostly water is used for them to bask in, and the entire description is fully applicable to these same forms, the main subject of this lecture. Same page, he says:

"Judging from Leibig's analysis of the active ingredient of the *yeast* cells, the *stoichiometric* constitution of the vibrionic sarcode is a nicety identical with that of the *muscular fibre* proper. In progress of the decomposition, the fibrin of the muscles seem to *dissolve*, as it were, substantially into those naked sarcode moleculus, which, however, are meat, find no material for forming into cells, or so-called yeast, for want of cellulosa."

They are, however, the true active principle, and *de facto* actually constitute the ferment of the yeast.

Here Leibig recognizes the same thing, and has, according to my ideas, guessed in them the true cause of fermentation, though only giving the naked statement, without collateral evidence to sustain his statements, which I shall attempt to clear up in some way.

"Hallier, Sanderson, Cheveau and others, have come to the conclusion that contagion of all zymotic diseases are constituted of living bodies or particles, and foresaw the idea that these solid organic bodies are micrococci, of specific fungoid plants." These are somewhat conjectural.

The yellow fever *microzymes* of authors, whether real or imaginary, I have no doubt are the identical organisms I am writing about.

Whether they propagate, as is imagined by a recent writer, by "seminal or sexual reproduction; by gamogenesis or by agamogenesis," I shall not attempt to settle at this time.

"Owen has advanced the name of *parthenogenesis*, among certain families of insects, as wholly independent of any male intercourse." This may possibly be doubted. "When food is no longer abundant, and weather no longer pleasant, she is no longer fruitful without the male, and in aphid life, *parthemogenesis* gives place to *gamogenesis.*

If it was not for fear of being thought mad, I would now predict that all the rocks and minerals, of every kind, on or in the earth, are more or less composed of them, and any rock, or any solid mineral substance, reduced to an impalpable powder, are chiefly composed of these living things, and they may play the main part in all chemical changes, including organic and inorganic chemistry, and that they are wholly indestructible, because so hard and so minute, that they cannot be reached so as to destroy or affect them. It might, at present, be going a little beyond human conceptions, to even hint that even solid metals are composed of them. That startling idea may turn out to be true, and our knowledge of natural laws will perhaps have to undergo still further advances. Even phvsiology may be better known, and therapeutics, too, will have to be studied under new aspects.

Mineralogy, crystalography, botany, pathology, relations of "force and matter," molecular and terrestrial magnetism, all may be better understood.

I am not sure that those life-motions, after all, may not be entirely magnetic, and governed by a law of polarity, as they seem to have a magnetic influence on each other, as they butt together, and are then suddenly repelled, as if by a shock; they may, notwithstanding, have organs of volition.

Some fifty other specimens besides those given, I had examined dry dust, and thought they were nothing but dust atoms. I find so far as I have examined them in water, to be identical with all the others, including metals, oxydes, salts, quartz, emery, and diamond dust—"several metals examined," with uniform results.

From the measurements made by myself, as well as by others, one cubic inch of these forms, of the smallest size, would amount to 125,000,000,000; the largest, 1,000,000,000, and the intermediate size, 31,250,000,000—numbers so vast that the mind becomes confused in the contemplation of them.

All specimens so far examined, any microscopist can easily prepare, and see for his own satisfaction, by following the plans given in this paper, if he has a good microscope—500 powers being favorable for the purpose, which is 250,000 superfices, or 125,000 in bulk.

The finer any solid is powdered the better; in fact, all solids seem to be composed wholly of them, when reduced to an impalpable powder. These little organisms cannot be impaired by triturating any length of time—they are indestructible. I have now come to the conclusion that they are not formed at all by fermentation, and that they do not grow or change in size, and I may even dream that they have existed just the same from all eternity. The plants must derive them through their roots in water, and animals derive them from plants ready formed, as they come from the rocks of ages.—*Nashville Jour. of Med. and Surg.*

